# Diagnostic value of SHOX2, RASSF1A gene methylation combined with CEA level detection in malignant pleural effusion

**DOI:** 10.1186/s12890-023-02462-z

**Published:** 2023-05-08

**Authors:** Shaosen Chen, Kunlun Huang, Lin Zou, Lu Chen, Peicun Hu

**Affiliations:** 1 Department of Respiratory and Critical Care Medicine, The Second People’s Hospital of Foshan, Foshan, 528000 China; 2 Clinical Laboratory, The Second People’s Hospital of Foshan, Foshan, 528000 China

**Keywords:** SHOX2, RASSFF1A, Methylation, CEA, Pleural effusion

## Abstract

**Aim:**

To investigate the diagnostic value of combined detection of SHOX2 and RASSF1A gene methylation with carcinoembryonic antigen (CEA) level in diagnosing malignant pleural effusion.

**Methods:**

Between March 2020 and December 2021, we enrolled 68 patients with pleural effusion admitted to the Department of Respiratory and critical care medicine of Foshan Second People's Hospital. The study group included 35 cases of malignant pleural effusion and 33 cases of benign pleural effusion. Methylation of the short homeobox 2 genes (SHOX2) and RAS-related region family 1A gene (RASSF1A) in pleural effusion samples were detected by real-time fluorescence quantitative PCR, and the level of carcinoembryonic antigen (CEA) in pleural effusion samples was detected by immune flow cytometry fluorescence quantitative chemiluminescence.

**Results:**

SHOX2 or RASSF1A gene methylation was detected in 5 cases in the benign pleural effusion group and 25 patients in the malignant pleural effusion group. The positive rate of SHOX2 or RASSF1A gene methylation in the malignant pleural effusion group was significantly higher than in the benign pleural effusion group (71.4% vs. 15.2%, *P* < 0.01). Positive CEA (CEA > 5 ng/m) was detected in 1 case in the benign pleural effusion group and 26 patients in the malignant pleural effusion group. The CEA-positive rate in the malignant pleural effusion group was significantly higher than in the benign pleural effusion group (74.3% vs. 3%, *P* < 0.01). When SHOX2 and RASSF1A gene methylation was combined with CEA detection, 6 cases were positive in the benign pleural effusion group, and 31 patients were positive in the malignant pleural effusion group. The positive rate of combined detection in the malignant pleural effusion group was significantly higher than in the benign pleural effusion group (88.6% vs. 18.2%, *P* < 0.01). The sensitivity, specificity, accuracy, positive predictive value, negative predictive value, and Youden’s index of SHOX2, RASSF1A gene methylation combined with CEA in diagnosing malignant pleural effusion were 88.6%, 81.8%, 85.3%, 83.8%, 87.1% and 0.7 respectively.

**Conclusion:**

The combined detection of SHOX2 and RASSF1A gene methylation with CEA level in pleural effusion has a high diagnostic value for malignant pleural effusion.

Pleural effusion is a common clinical problem affecting approximately 1.5 million patients each year [1]. It can be caused by various factors, including malignant pleural effusion (MPE), tuberculous pleural effusion, parapneumonic effusion, heart failure, and others [2]. The underlying cause of the effusion determines the treatment strategy; distinguishing benign from malignant pleural effusions is key [3]. The gold standards for diagnosing MPE are the cytological examination of pleural effusion and thoracoscopic pleural biopsy [2]. But both methods have limitations, including low sensitivity and sampling errors [4]. The specificity of thoracoscopic pleural biopsy is also close to 100%. Still, it is prone to sampling errors, is highly subjective, and has the risk of trauma and surgery-related complications [5]. Besides, both methods could report false negative results due to heterogeneity of MPE or sampling errors. Therefore, finding a more objective and sensitive diagnostic index of malignant pleural effusion is necessary. The detection of tumor biomarkers is a commonly used biological indicator to assist in the diagnosis of malignant pleural effusion. In recent years, the research and clinical application of representative tumor markers such as squamous cell carcinoma antigen (SCCAG), neuron-specific enolase (NSE), cytokeratin-19 fragment (CA211), carbohydrate antigen 153 (CA153), matrix metalloproteinases (MMPs) and carcinoembryonic antigen (CEA) are relatively deep and mature. SCCAG is used to diagnose malignant pleural effusion associated with squamous cell carcinoma. In diagnosing malignant pleural effusion, when the cut-off point of SCCAG is 0.65 ng/mL, the sensitivity is 72.3%, while the specificity is only 36.2% [6]. The sensitivity and specificity of NSE for malignant pleural effusion are 40.0% and 57.8%, which is also sued for diagnosing malignant pleural effusion associated with small cell lung cancer [7]. CA211 has significant clinical value in diagnosing malignant pleural effusion associated with lung squamous cell carcinoma, with strong specificity (80% ~ 91%) and sensitivity of only 55% ~ 60% [8]. CA153 increased in different degrees in malignant pleural effusion caused by lung cancer [8]. The study found that when CA153 > 77 IU/L, the sensitivity and specificity of diagnosis of malignant pleural effusion were 40% and 100% [9]. MMPs have a high diagnostic value for pleural effusion caused by MPM. The study found that the sensitivity and specificity of MMP-9 in predicting the malignant degree of pleural effusion were 95% and 73%, respectively (cut-off point > 639 ng/ml; AUC = 0.8) [10]. Detecting carcinoembryonic antigen (CEA) in pleural effusion is an effective auxiliary diagnostic indicator for malignant pleural effusion. However, it still has the limitation of low sensitivity (sensitivity 68%, specificity 95%) [7, 9]. Throughout the previous studies, because of the different pathological types of tumors, these tumor markers still lack high sensitivity or specificity in the diagnosis of malignant pleural effusion, and there are still some limitations in the diagnosis of malignant pleural effusion solely relying on the level of tumor markers in pleural effusion. DNA methylation has been a valuable tumor biomarker in recent years. The cytosine methylation in the CpG dinucleotide environment plays a crucial role in essential biological processes and human diseases. Abnormal DNA methylation is a sign of human cancer [11]. Among the known DNA methylation, studies have shown that SHOX2 is a promising biomarker for diagnosing malignant pleural effusion [12]. A meta-analysis of SHOX2 gene promoter methylation and lung cancer showed that the SHOX2 gene promoter methylation rate increased in lung cancer tissue, suggesting that SHOX2 methylation was significantly related to lung cancer [13, 14]. RASSF1A is considered a new tumor suppressor gene, closely associated with the occurrence of various malignant tumors [15]. As a tumor suppressor gene, the RASSF1A promoter hypermethylation rate reached 100% and 63% in small and non-small cell lung cancer, respectively [11, 16]. Based on the close relationship between SHOX2 and RASSF1A gene methylation and the occurrence of lung cancer, and MPE is mostly secondary to malignant tumors from other sources, mainly lung cancer, it is not clear whether SHOX2 and RASSF1A gene methylation status in pleural effusion, whether their methylation is related to malignant pleural effusion, and whether gene methylation in pleural effusion combined with tumor marker CEA detection can improve the diagnostic efficiency of malignant pleural effusion. This study aimed to evaluate the value of DNA methylation combined with CEA level in diagnosing malignant pleural effusion by detecting SHOX2, RASSF1A gene methylation, and CEA level in pleural effusion.

## Materials and methods

### Patients

The clinical data of 68 patients with PE admitted to the Department of Respiratory and critical care medicine of Foshan Second People's Hospital from March 2020 to December 2021 were retrospectively analyzed. Demographic characteristics were recorded. Diagnostic criteria of MPE: malignant cells were found in pleural effusion cytology or pleural tissue biopsy specimens. Diagnostic criteria for benign pleural effusion (BPE): (1) no tumor cells were found in pleural effusion or pleural biopsy; (2) The pleural effusion disappeared without recurrence after etiological treatment and thoracentesis; (3) No tumor was diagnosed during the half year follow-up. In addition, pleural effusion and pleural tissue were also sent for cell and histopathological examination. The pathological diagnosis of tissue and cytological specimens was made by two or more pathologists independently.

### Detection of SHOX2, RASSF1A gene methylation, and CEA in pleural effusion

After obtaining informed consent, all patients underwent thoracentesis and/or percutaneous pleural biopsy or thoracoscopic pleural biopsy, and pleural effusion and/or pleural tissue samples were collected. Pleural effusion was sent to the medical laboratory for SHOX2, RASSF1A gene methylation, and CEA level detection. The methylation of short homeobox 2 gene (SHOX2) and RAS-related region family 1A gene (RASSF1A) in pleural effusion was detected by real-time fluorescence quantitative PCR following these experimental methods: human SHOX2, RASSF1A gene methylation DNA detection kit (PCR fluorescence method) was purchased from Shanghai Thorough Life Technology Co., Ltd. Steps: (1) DNA extraction: first take 10 ml of pleural effusion, centrifuge at 2000 rpm for 10 min, and remove the supernatant and then use the remaining cell precipitation for DNA extraction. The genomic DNA extraction kit (centrifugal column type) of body fluid/cell/tissue of Tiangen Biochemistry was used to extract DNA from cell precipitation. (2) Sulfite modification: take 20ul of extracted DNA for sulfite modification. The EZ DNA Method-DirectTM Kit of ZYMO RESEARCH Biological Company was adopted. Please operate the sulfite modification process strictly following the kit’s instructions, and the modified DNA will be used for detection immediately. (3) DNA methylation detection: prepare the mixture of the PCR reaction solution and DNA polymerase according to the instructions; take 15ul of the mixture and add it into the PCR reaction tube, and then add 5ul of DNA of the sample to be tested after sulfite modification. At the same time, set the quality control sample and negative control, mark it, mix it upside down, and centrifuge it instantaneously. Put the PCR reaction tube into the American ABI7500 detection system. Reaction conditions: 95 ℃, 10 min, 1 cycle; 95 ℃, 15 s, 60 ℃, 30 s, 5 cycles; 95 ℃, 15 s, 57 ℃, 30 s, 40 cycles. (4) Positive criteria: a. The FAM fluorescence signal amplification curve is smooth 'S' shape, and the Ct value is less than 35, indicating that RASSF1A gene methylation is positive. B. The amplification curve of the VIC (or HEX) fluorescence signal is smooth 'S' shape, and the Ct value is less than 32, indicating that SHOX2 gene methylation is positive.

The level of CEA in pleural effusion was detected by chemiluminescent particle immunoassay. The reagent was purchased from Abbott Ireland Diagnostics, and the instrument was an Abbott ARCHITECT I200 chemiluminescence analyzer. The usual range of CEA in pleural effusion is set to 0-5 ng/ml, and CEA > 5 ng/ml is positive.

### Statistical analysis

Data were analyzed by χ 2 analysis; The sensitivity, specificity, accuracy, positive predictive value, negative predictive value, and Youden’s index of tumor biomarkers for single and combined diagnosis of malignant pleural effusion were calculated. Spss19.0 software was used for statistical analysis, and *p* < 0.05 was statistically significant.

## Results

### Patient characteristics

Sixty-eight eligible patients were included in the study and analyzed retrospectively, including 33 cases in the benign pleural effusion group, 25 males and 8 females, with an average age of 70 ± 17 years. The leading causes include heart failure, hypoalbuminemia, tuberculous pleurisy, and pneumonia-like pleural effusion. There were 35 cases in the malignant pleural effusion group, including 14 males and 21 females, with an average age of 74 ± 11 years. The leading causes included lung cancer with pleural metastasis and non-lung tumors with pleural metastasis, as shown in Table 1.

**Table 1:** Patient general characteristic

Group	BPE (n)	MPE (n)
Gender( male, female)	33(25/8)	35(14/21)
Age	70 ± 17	74 ± 11
Cause composition		
Heart failure	9	
Hypoproteinemia	4	
Tuberculous pleurisy	14	
Bacterial pneumonia	6	
Adenocarcinoma		23
Squamouscarcinoma		1
SCLC		1
NSCLC		10

### Comparison of positive rates of SHOX2 and RASSF1A gene methylation between benign and malignant pleural effusion groups

SHOX2 or RASSF1A gene methylation was detected in 5 of 33 benign pleural effusions. The positive rate of SHOX2 and RASSF1A gene methylation was 15.2%; SHOX2 or RASSF1A gene methylation was detected in 25 of 35 cases of malignant pleural effusion. The positive rate of SHOX2 and RASSF1A gene methylation was 71.4%; The positive rates of SHOX2 and RASSF1A gene methylation in the malignant pleural effusion group were significantly higher than those in the benign pleural effusion group (*p* < 0.01), as shown in Table 2.

**Table 2:** Comparison of positive rates of SHOX2 and RASSF1A methylation in benign and malignant pleural effusion groups

Group	Total (n)	Methylation positive (n)	Methylation postive rate	*p*
BPE	33	5	15.2%	< 0.01
MPE	35	25	71.4%	

### Comparison of CEA positive rate (CEA +) between benign and malignant pleural effusion groups

**Fig. 1:**
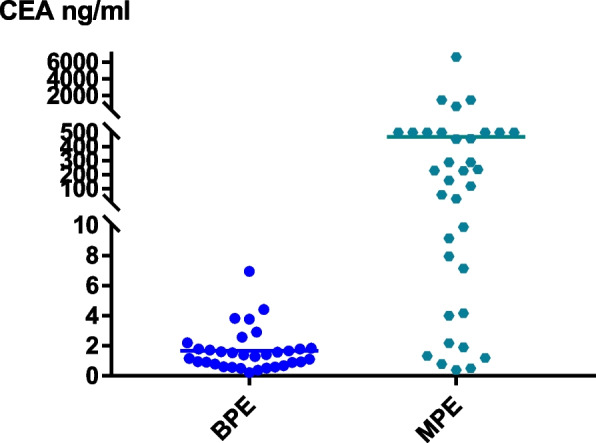
Distribution of CEA expression level in benign and malignant pleural effusion

The expression level of CEA in the benign and malignant pleural effusion groups is significantly different. The expression level of CEA in the malignant pleural effusion group is significantly higher than in the benign pleural effusion group (Fig. 1). CEA positive is defined as > 5 ng/ml. Only 1 of 33 benign pleural effusions were CEA + , and the CEA positive rate was 3% in the BPE group; Among the 35 cases of malignant pleural effusion, 26 cases were CEA + , and the CEA positive rate was 74.3% in the MPE group. The CEA positive rate in the MPE group was significantly higher than that in the BPE group (*p* < 0.01), as shown in Table 3.

**Table 3:** Comparison of CEA positive rates between benign and malignant pleural effusion groups

Group	Total (n)	CEA positive (n)	CEA positive rate(%	*p*
BPE	33	1	3	< 0.01
MPE	35	26	74.3	

### Comparison of positive rates of SHOX2, RASSF1A gene methylation combined with CEA in benign and malignant pleural effusion groups

Taking the positive results of SHOX2, RASSF1A gene methylation, and CEA as the positive criteria of combined detection, DNA methylation or CEA were detected in 6 cases of benign pleural effusion group, and the positive rate was 18.2%; SHOX2 and RASSF1A gene methylation or CEA were detected in 31 cases of malignant pleural effusion group, with a positive rate of 88.6%. The positive rate of SHOX2 and RASSF1A gene methylation combined with CEA in the malignant pleural effusion group was significantly higher than in the benign pleural effusion group (*p* < 0.01), as shown in Table 4.

**Table 4:** Comparison of positive rates of SHOX2, RASSF1A methylation combined with CEA in benign and malignant pleural effusion group

Group	Total (n)	Combined positive (n)	combined positive rate(%)	*p*
BPE	33	6	18.2	< 0.01
MPE	35	31	88.6	

### The value of different biomarkers in the diagnosis of malignant pleural effusion

**Fig. 2:**
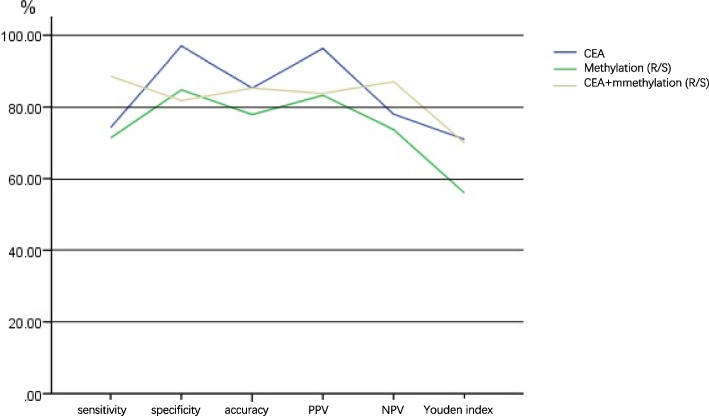
Evaluation of the diagnostic efficacy of different biomarkers in malignant pleural effusion

The sensitivity, specificity, accuracy, positive predictive value, negative predictive value, and Youden’s index of SHOX2, RASSF1A gene methylation, CEA, and their combined detection in pleural effusion for the diagnosis of malignant pleural effusion were calculated, respectively. The results showed that SHOX2 and RASSF1A gene methylation combined with CEA detection in pleural effusion had higher sensitivity and specificity, accuracy, and Youden’s index for diagnosing malignant pleural effusion, as shown in Table 5 and Fig. 2.

**Table 5:** Diagnostic value of different biomarkers in malignant pleural effusion

Group	CEA	Methylation	Combined
Sensitivity(%)	74.3	71.4	88.6
Specificity(%)	97.0	84.8	81.8
Accuracy(%)	85.3	77.9	85.3
PPV (%)	96.3	83.3	83.8
NPV (%)	78.0	73.7	87.1
Youden’s index	0.71	0.56	0.70

## Discussion

Malignant pleural effusion is caused by pleural metastasis of a malignant or primary malignant tumor in the pleura. It is one of the common complications of malignant tumors. To determine whether malignant pleural effusion will involve staging and treating the disease. Traditional cytopathology is the gold standard for diagnosing malignant pleural effusion, but its sensitivity could be more satisfying. It is an unmet need to find efficient biomarkers to assist in the diagnosis of malignant pleural effusion.

The essence of cancer is the malignant proliferation and proliferation of cells, and the epigenetic mechanism is mainly about the specific expression of genes and the normal growth and development of individuals [17]. DNA methylation is one of the epigenetic modifications of genes. 5-methylcytosine (5mC) and 5-hydroxymethylcytosine (5hmC) are the most studied base modifications. 5mC and 5hmC affected the expression of related genes in tumor cells through hypermethylation of tumor suppressor genes and hypomethylation of carcinogenic genes. SHOX2 gene is a member of the homeobox gene family, which is widely described as a transcription regulator and is closely related to organ development [14]. In 2010, Schmidt et al. found for the first time that SHOX2 methylation can distinguish benign and malignant lung lesions (sensitivity 68%, specificity 95%) [12]. Ilse et al. found high methylation of the SHOX2 gene in pleural effusion samples, with a specificity of 96.2% and sensitivity of 39.5% [18]. A meta-analysis showed that SHOX2 methylation had higher sensitivity (70%) and specificity (96%) than carcinoembryonic antigen and cytokeratin 19 fragment antigen 21–1 in MPE associated with non-small cell lung cancer [19]. RASSF1A is a part of the Ras signal pathway, which mainly regulates cell proliferation and apoptosis and belongs to the tumor suppressor gene. In 2012, Fujii et al. detected specific DNA methylation status in the pleural effusion of patients with different lung diseases (malignant mesothelioma, lung cancer, asbestos pleurisy). The results showed that abnormal methylation of the RASSF1A promoter in pleural effusion might be a valuable marker for distinguishing benign and malignant pleural effusion [20]. In this study, we selected 68 patients with pleural effusion and divided them into benign and malignant pleural effusion groups after being confirmed by cytology or histopathology. The causes of the benign pleural effusion group include heart failure, hypoproteinemia, tuberculous pleurisy, bacterial pneumonitis, and other causes of pleural effusion; The etiology of malignant pleural effusion group includes lung cancer-related malignant pleural effusion and pleural effusion caused by a non-pulmonary malignant tumor. We detected the methylation of the SHOX2 gene and RASSF1A gene in two groups of pleural effusion. We found that the positive rate of SHOX2 gene and/or RASSF1A gene methylation in benign pleural effusion was 15.2%.

In contrast, the positive rate of SHOX2 gene and/or RASSF1A gene methylation in malignant pleural effusion was 71.4%. The methylation of SHOX2 and RASSF1A genes in malignant pleural effusion was significantly higher than in benign pleural effusion. The sensitivity, specificity, accuracy, positive predictive value, negative predictive value, and Youden’s index of SHOX2 and RASSF1A gene methylation detection in diagnosing MPE were 71.4%, 84.8%, 77.9%, 83.3%, 73.7% and 0.56, respectively. Our research is consistent with previous relevant research results. Katayama H et al. studied the methylation of five tumor suppressor genes, such as MGMT, p16INK4a, and RARb, in pleural effusion. In the expression of malignant pleural effusion, it was found that in patients with malignant pleural effusion, patients with hypermethylation of MGMT, p16 (INK4a), RASSF1A, or RARbeta were 5.68 times higher than those without methylation (*P* = 0.008), suggesting that abnormal methylation of gene promoter was more common in patients with malignant pleural effusion than in patients with benign pleural effusion [21]. Dietrich D conducted cytological and DNA methylation analysis in a case–control study, including PE of 114 patients (58 cases, 56 controls). Cytological analysis and SHOX2 and SEPT9 methylation results obtained 100% specificity, but the overall positive rate of SHOX2 and SEPT9 was 26%. The study confirmed the role of SHOX2, SEPT9, and WIF-1 promoter methylation in diagnosing MPE, but the sensitivity of diagnosis still needs to be improved [22]. The above studies show that detecting SHOX2 and RASSF1A gene methylation in pleural effusion has a high diagnostic value for malignant pleural effusion and can be used as a new biological indicator for the clinical diagnosis of malignant pleural effusion.

Tumor markers are commonly used biomarkers to assist in the diagnosis of malignant pleural effusion. They are the most widely studied indicators used to diagnose malignant pleural effusion. So far, some studies on biomarkers that distinguish malignant pleural effusion from benign pleural effusion have been reported, such as CEA, carbohydrate antigen 125 (CA125), vascular endothelial growth factor (VEGF), interleukin 17 (IL-17), soluble mesothelin-related peptide (SMRP). These studies found that CA125 was positive in 26%—44% of lung cancer patients, and the positive rate was positively correlated with the stage and progression of lung cancer [23]. VEGF mainly participates in the proliferation of vascular endothelial cells, increases vascular permeability, and participates in cancer cell migration. The increase in vascular permeability is the direct reason for the formation of MPE, and the promotion of angiogenesis is the indirect reason. A total of 540 patients in five studies were included in the mean difference analysis of VEGF level in pleural effusion, and the results showed that it was higher than BPE in MPE [9]. When the level of IL-17 in MPE patients increases, and IL-17 is less than 15 pg/mL, the survival time of lung cancer patients is prolonged, which may be an indicator for the diagnosis and prediction of lung cancer with MPE [24]. SMRP exists in normal serous mesothelial cells and is overexpressed in various cancers. Many studies have shown that the level of SMRP in serum and pleural effusion has a vital reference value for diagnosing MPM. There is no significant difference between the two types of samples, and it can monitor the progress of the disease and the response to drug treatment [25]. Among these biomarkers, CEA is considered the most valuable indicator for auxiliary diagnosis of malignant pleural effusion. CEA is a polysaccharide-protein complex located on the surface of tumor cells. It is one of the earliest markers used in malignant tumors—the serum CEA level increases in multiple organ tumors, including primary lung cancer [7]. The concentration of CEA in the serum of healthy people is extremely low. When a cell becomes cancerous, its suppressed genes are reactivated, and tumor cells synthesize and secrete CEA, which is difficult to enter the blood circulation because of its large molecular weight. Therefore, the concentration of CEA in malignant pleural effusion increased earlier and more significantly than in serum [26]. According to the literature, the sensitivity of CEA in diagnosing MPE is low, about 25%—57%, and the specificity is 90%—100% [27]. This study detected the CEA level in benign and malignant pleural effusions. We found that the proportion of the abnormal increase in CEA level in malignant pleural effusion was significantly higher than in benign pleural effusion (74.3% vs. 3%). When CEA > 5n/ml of pleural effusion was taken as the diagnostic threshold, the sensitivity, specificity, accuracy, positive predictive value, negative predictive value, and Youden’s index of CEA in diagnosing MPE was 74.3%, 97.0%, 85.3%, 96.3%, 78.0% and 0.71 respectively. Our research results are similar to previous related studies, indicating that CEA has high efficiency in diagnosing malignant pleural effusion, especially with high specificity. At the same time, in our study, the sensitivity of CEA in diagnosing malignant pleural effusion is as high as 74.3%, which seems to be higher than the previous research results. We believe this is related to the fact that most of the subjects we studied are malignant pleural effusions associated with adenocarcinoma. CEA has a high diagnostic sensitivity in malignant pleural effusions associated with adenocarcinoma.

As in previous studies, it may be affected by the heterogeneity of pathological tumor types. Whether DNA methylation or tumor markers, a single biological indicator is not ideal for diagnosing malignant pleural effusion, combined detection of multiple biological indicators can often improve the sensitivity of diagnosis. This study analyzed the efficacy of SHOX2 and RASSF1A gene methylation combined with CEA detection in diagnosing malignant pleural effusion. The results showed that SHOX2/RASSF1A gene methylation combined with CEA detection in the diagnosis of malignant pleural effusion had 88.6%, 81.8%, 85.3%, 83.8%, 87.1%, and 0.7 of sensitivity, specificity, accuracy, positive predictive value, negative predictive value, and Youden’s index, respectively. Compared with single index evaluation, this method improves the sensitivity of diagnosis of malignant pleural effusion, reduces the missed diagnosis rate, and ensures reasonable specificity and accuracy. Therefore, SHOX2 and RASSF1A gene methylation combined with CEA detection may be a suitable prediction combination for diagnosing MPE, which can improve the clinical diagnostic efficiency of MPE.

This study preliminarily discussed the diagnostic value of SHOX2 and RASSF1A gene methylation combined with CEA detection for malignant pleural effusion. Due to the small sample size, this study also has some limitations. First, most of the malignant pleural effusion group are malignant pleural effusions associated with adenocarcinoma. It is still being determined whether there is a DNA methylation difference in different pathological types of malignant pleural effusion. Secondly, DNA methylation level is only qualitative detection, not quantitative statistical analysis. In the future, we will further verify our research conclusions by expanding the sample size, conducting a hierarchical analysis of the DNA methylation status of malignant pleural effusion of different pathological types, and conducting a quantitative analysis of the DNA methylation level.

## Data Availability

The datasets generated during and/or analyzed during the current study are available from the corresponding author upon reasonable request.
